# Joint Model Partitioning and Bandwidth Allocation for UAV-Assisted Space–Air–Ground–Sea Integrated Network: A Hybrid A3C-PPO Approach

**DOI:** 10.3390/e28030337

**Published:** 2026-03-18

**Authors:** Yuanmo Lin, Yuanyuan Han, Minmin Wu, Shaoyu Lin, Xia Zhang, Zhiyong Xu

**Affiliations:** 1 College of Artificial Intelligence, Putian University, Putian 351100, China; linyuanmo@ptu.edu.cn (Y.L.); 202580221101@stu.ptu.edu.cn (Y.H.);; 2 College of Communications Engineering, Army Engineering University of PLA, Nanjing 210000, China; 3 College of Computer and Data Science, Putian University, Putian 351100, China

**Keywords:** space–air–ground–sea integrated network (SAGSIN), unmanned aerial vehicle (UAV), mobile edge computing, deep neural network, model partitioning

## Abstract

Unmanned Aerial Vehicle (UAV)-assisted mobile edge computing is pivotal for the Space–Air–Ground–Sea Integrated Network (SAGSIN) to support heterogeneous task offloading. However, the inherent resource constraints of UAVs limit their ability to support intensive and concurrent task processing in dynamic environments. In such complex scenarios, the dual requirements of discrete model partitioning and continuous bandwidth allocation make it difficult for traditional reinforcement learning algorithms to achieve optimal resource matching. Therefore, in this paper, we design a joint optimization framework based on Asynchronous Advantage Actor-Critic (A3C) and proximal policy optimization (PPO). Specifically, the model partitioning strategy is learned through PPO, which utilizes a clipped objective function to ensure training stability and generalization across complex Deep Neural Network (DNN) structures. Moreover, the framework leverages the asynchronous multi-threaded architecture of A3C to dynamically allocate bandwidth, effectively accommodating rapid fluctuations in terminal access. Finally, to prevent resource monopolization and ensure fairness, a weighted priority scheduling mechanism based on task urgency and computation time is introduced. Extensive simulations show that the proposed algorithm outperforms existing approaches in terms of task completion rate, task processing latency, and resource utilization under dynamic SAGSIN scenarios.

## 1. Introduction

The rapid evolution and large-scale deployment of 5G technology provide critical support for intelligent upgrades across various complex scenarios. Key enabling characteristics of 5G [1] include large bandwidth, low latency, and high reliability. The Space–Air–Ground–Sea Integrated Network (SAGSIN) serves as a key architecture for overcoming coverage limitations of conventional single-layer networks. Continuous global network coverage is achieved in SAGSIN through integration of space-based satellites, aerial platforms, terrestrial nodes, and maritime communication facilities. SAGSIN has been widely adopted in critical domains such as intelligent transportation, emergency rescue, ocean monitoring, and remote services [2]. Computation tasks generated by various terminal devices in SAGSIN scenarios exhibit heterogeneous and intensive characteristics. Heterogeneous and intensive task characteristics impose stringent requirements on network computing capability and service response efficiency. Conventional centralized cloud computing can no longer adequately satisfy the demands of scenario-specific services [3].

To mitigate the inadequacies of centralized cloud computing in meeting scenario-specific service demands, Mobile Edge Computing (MEC) has been introduced by deploying computing resources at the network edge [4]. Moreover, the MEC in SAGSIN confronts unique challenges attributed to heterogeneous access methods and dynamic topology across the space, air, ground, and sea domains [5]. The global coverage capability of SAGSIN, coupled with its complex deployment environments, imposes significant practical limitations on ground-based MEC protocols. Ground edge nodes are typically constrained by geographical conditions, which render effective coverage of remote areas, dynamic scenarios, and temporary task regions challenging to achieve. Furthermore, high deployment costs and inadequate flexibility hinder the ability of these ground edge nodes to adapt to dynamic shifts in task distribution. Unmanned Aerial Vehicles (UAVs), as aerial platforms featuring high mobility and flexible deployment [6], are well-aligned with the requirements of SAGSIN scenarios. The integration of UAVs into the MEC framework gives rise to a UAV-assisted MEC paradigm, through which on-demand and rapid networking is enabled. UAV edge nodes dynamically adjust their deployment positions based on task distribution [7], providing proximity-based computation offloading services to terminal devices. Through such dynamic adjustments, task transmission distances and processing latency are effectively reduced, and coverage gaps of ground edge nodes are addressed. Consequently, the global coverage capability of MEC services in SAGSIN scenarios is significantly improved.

Resource constraints of UAVs constitute the primary factor that degrades service quality in UAV-assisted MEC paradigms [8]. Payload limitations of UAVs restrict the computing resources, communication bandwidth, and energy resources available on board. Dense heterogeneous tasks in SAGSIN scenarios demand differentiated resource requirements. Traditional full-task offloading modes easily cause resource allocation imbalance, task blocking, or degradation of service quality. Model partitioning techniques divide complex Deep Neural Networks (DNNs) into multiple sub-models according to specific rules [9]. The efficiency of this paradigm under limited UAV computing power stems from the inherent data compression property of early DNN layers. As raw input propagates through initial convolutional and pooling layers, its dimensionality is progressively reduced, generating intermediate feature maps that are significantly smaller than the original data [2]. By executing these front layers locally, terminals avoid transmitting voluminous raw data, which drastically reduces communication overhead while alleviating the computational burden on resource-constrained UAVs. Flexible adjustments are enabled based on the resource availability of UAVs and task characteristics. Sub-models are reasonably allocated and collaboratively executed between terminal devices and UAV edge nodes. Resource load pressure on individual nodes is thereby effectively reduced. In this context, optimized resource allocation for UAV edge nodes is designed. Model partitioning strategies are integrated to achieve efficient matching between resources and tasks. Crucially, bandwidth allocation and model partitioning are tightly coupled and exhibit bidirectional dependencies. The allocated bandwidth strictly determines the transmission rate, which in turn dictates the transmission latency at different candidate partitioning points. Conversely, the chosen partitioning point determines the volume of intermediate data to be offloaded, altering the bandwidth demands. Independent optimization of either subproblem fundamentally ignores this coupling, inevitably leading to globally suboptimal performance. Therefore, a joint optimization framework is imperative. This approach serves as a critical pathway to enhance the overall performance of UAV-assisted MEC systems in SAGSIN scenarios.

## 2. Related Work

In UAV-assisted MEC systems within SAGSIN scenarios, the joint optimization of model partitioning and bandwidth allocation constitutes the core mechanism for enhancing computation offloading performance [10]. Reinforcement learning algorithms serve as the key technological enabler in this domain due to their adaptive decision-making capability in dynamic environments [11,12]. Significant differences exist in the scenario adaptability of various reinforcement learning algorithms for model partitioning tasks. The Deep Q-Network (DQN) algorithm is limited by its discrete action space [13]. Consequently, DQN is suitable only for model partitioning in linear sequential structures. Complex DNN models with multi-branch or nested topologies cannot be effectively handled by DQN. Algorithms such as Deep Deterministic Policy Gradient (DDPG) [14] and Twin Delayed DDPG (TD3) [15] focus on control requirements in continuous action spaces. These algorithms perform well in scenarios like robotic motion and vehicle speed regulation. However, the dual requirements of discrete decisions and continuous parameter adjustments in model partitioning cannot be simultaneously satisfied by these algorithms.

The proximal policy optimization (PPO) algorithm is built on the Actor-Critic framework [16]. PPO flexibly accommodates both discrete and continuous action spaces. Linear sequential model partitioning requirements are met. Complex topological model partitioning decisions are efficiently handled. The generalization capability of PPO significantly outperforms that of comparable algorithms. PPO imposes constraints on policy update magnitude through its proximal policy optimization mechanism. Training instability and divergence issues in traditional policy gradient algorithms, such as Advantage Actor-Critic (AC) and A2C [17,18], are effectively resolved. Hyperparameter tuning costs of PPO remain extremely low. Complex parameter debugging is not required. Convergence to the global optimal partitioning point is usually achieved with only a few iterations. PPO employs a sample reuse mechanism, where samples are collected once and reused multiple times for policy updates. Computational resource consumption of UAVs is thereby substantially reduced. The resource-constrained characteristics of UAVs are perfectly matched by this approach.

Bandwidth allocation constitutes another core component in UAV-assisted MEC within SAGSIN scenarios [19]. Stringent requirements are imposed on the dynamic adaptability and computational efficiency of algorithms, in alignment with the optimization needs of model partitioning. The unique characteristics of the Asynchronous Advantage Actor-Critic (A3C) algorithm render it the preferred solution for this task [20]. A multi-threaded asynchronous training architecture is adopted by the A3C algorithm. Multiple parallel actor networks independently interact with the environment to collect data. Gradient information is aggregated to the central network for parameter updates. Policy iteration is achieved without waiting for any single thread to complete a full trajectory sample. Convergence speed is significantly accelerated. Computational load on individual threads is reduced. These features align closely with the limited computational resources of UAVs.

The asynchronous training mechanism of A3C enables the synchronous collection of interaction experiences across diverse scenarios [21]. Specifically, experiences are collected from scenarios featuring different numbers of terminal connections, bandwidth occupancy rates, and transmission requirements. The dynamic characteristics of the bandwidth allocation environment in SAGSIN scenarios are comprehensively captured, enabling effective handling of terminal access fluctuations and heterogeneous bandwidth demands. Consequently, the resulting bandwidth allocation policies achieve both real-time responsiveness and allocation efficiency.

Several prior works have explored hybrid DRL models for joint optimization in MEC systems. Ahmed et al. [22]. employed a single DRL agent for joint task offloading and bandwidth allocation in multi-user MEC, but its unified action space fails to decouple heterogeneous decision structures with different update frequencies. Xu et al. [23]. combined DDQN and DDPG for joint resource allocation in MEC-assisted Railway IoT, yet it relies on centralized training without asynchronous exploration and does not address DNN partitioning. Xue et al. [24]. proposed DDPQN, integrating DDPG and DQN within a single policy network for DNN offloading, but its tightly coupled action representation limits independent subproblem optimization.

This work aims to address the dual optimization requirements of model partitioning and bandwidth allocation in UAV-assisted MEC systems under SAGSIN scenarios. No single algorithm can simultaneously meet the core demands of both tasks [25], algorithms specialized in model partitioning often lack the real-time performance and distributed training capabilities necessary for dynamic bandwidth allocation, while those proficient in bandwidth allocation suffer from deficiencies in generalization and training stability when dealing with complex model partitioning. Notably, in model partitioning tasks, the PPO algorithm demonstrates strong generalization capability, stable training performance, and high sample efficiency. In contrast, the Asynchronous Advantage Actor-Critic (A3C) algorithm exhibits fast convergence and excellent adaptability to dynamic environments in bandwidth allocation tasks. These two algorithms thus offer complementary advantages that can be synergistically leveraged. To fully exploit these complementary strengths, this paper proposes an A3C-PPO-based joint optimization method for bandwidth allocation and DNN partitioning. Specifically, PPO is tasked with optimizing model partitioning decisions, as it can precisely match the requirements of complex DNN partitioning while effectively accommodating the computational constraints of UAVs. A3C handles dynamic bandwidth resource allocation and supports rapid adaptation to the time-varying conditions inherent in SAGSIN scenarios. The collaborative operation of these two algorithms forms a comprehensive optimization framework, which comprehensively addresses the core challenges in model partitioning and bandwidth allocation. Consequently, the overall performance of UAV-assisted MEC systems in SAGSIN scenarios is significantly enhanced.

To tackle the challenges mentioned above, this paper proposes a joint optimization approach for bandwidth allocation and DNN partitioning in UAV-assisted MEC. The proposed approach combines weighted priority scheduling with A3C-PPO. The main contributions of this paper are as follows:

1. A UAV-assisted MEC architecture is proposed to minimize task execution latency while maximizing completion rates in SAGSIN. Specifically, the framework addresses the resource constraints of intelligent terminals by leveraging UAV-mounted servers. The architecture decouples the complex non-convex optimization problem into two tractable subproblems, the bandwidth allocation strategy for UAVs and the DNN model partitioning for terminals.
2. A hybrid optimization framework integrating A3C and PPO is proposed for SAGSIN. Heterogeneous temporal dynamics and structural requirements of bandwidth allocation and DNN partitioning are explicitly recognized. High-frequency adaptation to channel fluctuations is handled by the asynchronous architecture of A3C. Stable topology-aware discrete decisions are governed by the clipped objective function of PPO. Rapid convergence in dynamic environments and robust generalization across model topologies are achieved. The scenario-driven algorithm assignment is fundamentally distinguished from generic decomposition strategies.
3. Based on the above framework, the proposed scheduling algorithm quantifies task priority by integrating computation time and remaining available time. Through the dynamic calculation of priority, urgent tasks are guaranteed execution resources, while short tasks are prioritized according to the Short-Job-First principle, avoiding resource monopolization by long-duration tasks that causes service timeouts.

The rest of this paper is organized as follows. Related work is reviewed in Section 2. The system model and problem formulation are presented in Section 3. The proposed algorithms are described in detail in Section 4. The effectiveness and adaptability of the proposed algorithms are evaluated through comparative experiments in Section 5. Section 6 concludes the paper and outlines future research directions. This version is concise, follows standard SCI paper organization phrasing, and maintains a formal yet clear tone.

## 3. System Modeling and Problem Formulation

In this section, we introduce a general task scheduling framework within SAGSIN. We first extensively construct the network model of SAGSIN and the channel model. Subsequently, we build the task model, model partitioning, and bandwidth allocation. The overall parameter symbols are shown in Table 1.

### 3.1. Network Model

**Figure 1:**
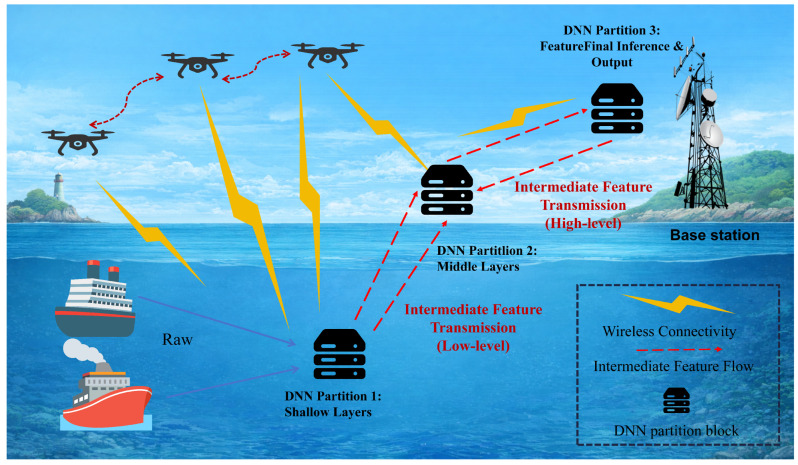
DNN Task Partitioning in UAV-Assisted MEC over SAGSIN.

As shown in Figure 1, the UAV-assisted SAGSIN network primarily consists of terminal devices and UAVs. UAVs fly from their initial positions to final positions and serve as edge servers to provide computational support for intelligent terminal devices. During the service cycle of each UAV, intelligent terminal device $v_{i}$ remains associated with the nearest UAV $u_{m}$. To ensure service quality in the UAV-assisted MEC network, the number of intelligent terminals connected to each UAV is strictly limited. Consequently, the required number of deployed UAVs depends on the number of intelligent terminals to be served.

The UAV-assisted SAGSIN network is represented by an undirected graph G=(U,V,E), where $U=\{u_{1},u_{2},\ldots ,u_{M}\}$ denotes the set of UAVs, and *M* is the number of UAVs. The maximum number of intelligent terminals that UAV $u_{m}$ can serve is denoted as $a_{m}$. $V=\{v_{1},v_{2},\ldots ,v_{N}\}$ denotes the set of intelligent terminals, and *N* is the number of intelligent terminals. $E=\{e_{1},e_{2},\ldots ,e_{N}\}$ represents the association relationships between intelligent terminals and UAVs.

The DNN tasks running on different intelligent terminals may belong to the same type or different types. Due to limited computational capability on intelligent terminals, direct local execution of compute-intensive DNN-based applications (e.g., image query) significantly increases processing time and introduces substantial latency. However, complete offloading of the entire DNN task to the UAV leads to high bandwidth consumption during transmission of large input-layer data, thereby increasing transmission latency and posing risks of user privacy leakage due to full transmission of sensitive data. To reduce processing latency of DNN tasks while preserving data privacy, intelligent terminals can collaboratively execute DNN tasks with the edge server. The DNN model is partitioned into two parts: the front part is executed locally on the intelligent terminal, and the intermediate feature results are transmitted to the UAV, where the remaining part is executed by the UAV of edge server.

Each UAV acts as an agent and determines the bandwidth allocation strategy and task offloading strategy for connected intelligent terminals based on the total available bandwidth, the number of connected intelligent terminals, and the structure of pending DNN models. After executing the decisions, the agent updates the bandwidth allocation and task offloading strategies according to the received rewards.

Assume that the DNN model corresponding to task $j_{i}$ on intelligent terminal $v_{i}$ consists of $L_{i}$ layers. The partitioning point is denoted as *k*, where the first *k* layers are executed on the edge device and the remaining $L_{i}-k$ layers are executed on the UAV, with *k* ranging from 0 to $L_{i}$. The execution of a DNN task is decomposed into four main stages. These stages include the execution latency of the first *k* layers on the edge device denoted as $T_{i,ed}^{k,q}$, the transmission latency from the edge device to the UAV denoted as $T_{i,tran}^{k}$, the execution latency of the remaining $L_{i}-k$ layers on the UAV denoted as $T_{i,uav}^{k}$, and the latency of returning computation results from the UAV to the terminal device. The result return latency is negligible due to the small size of the output data. Since tasks arrive continuously, task execution on the edge device requires priority-based queuing. Therefore, the execution latency of the first *k* layers on the edge device for task $j_{i}$ is given by

(1) $$T_{i,tot}^{k}=T_{i,ed}^{k,q}+T_{i,tran}^{k}+T_{i,uav}^{k}.$$

The optimization objective is to minimize the total processing latency of DNN tasks. Therefore, the optimal partitioning point *K* for task $j_{i}$ is determined by

(2) $$K=\arg\min_{k}T_{i,\mathrm{tot}}^{k}$$

### 3.2. DNN Task Latency Analysis

DNNs comprise multiple hidden layers, and model partitioning is often performed at the granularity of individual layers. Researchers typically analyze the computation latency of each layer to identify the optimal partitioning point. This analysis focuses on major layer types, including convolutional, pooling, and fully connected layers. However, the actual computation latency of a layer depends on input data size, its architectural type, and the hardware characteristics of the executing device. Although regression models are widely adopted to predict per-layer latency, they fail to account for inter-layer optimizations implemented in modern deep learning frameworks. Consequently, predicted latencies often deviate significantly from measured execution times. Because intelligent terminals generally execute only a limited set of DNN tasks, this paper adopts the real-time latency analysis method for specific DNN workloads proposed in [21].

The DNN task $j_{i}$ is executed on the intelligent terminal, and the computation latency of the first *k* layers $T_{i,ed}^{k}$ is recorded, where $k\in \{0,1,\ldots ,L_{i}\}$. Due to continuous task arrivals, task execution on the intelligent terminal requires priority-based queuing. The queuing waiting latency for DNN task $j_{i}$ on the ED is denoted as $T_{i,ed}^{q}$. Task arrivals at each intelligent terminal are modeled as a Bernoulli process, where terminal *i* independently generates a task in each time slot *t* with probability $\lambda_{i}$. The service time is deterministic, as the computational workload is fixed once the DNN partitioning point is determined [26]. Let $Q_{i}\left(t\right)$ denote the queue length of terminal *i* at the beginning of time slot *t*. The queue evolution is given by $Q_{i}\left(t+1\right)=\max\left(0,\;Q_{i}\left(t\right)+a_{i}\left(t\right)-\mu_{i}\left(t\right)\right),$ where $a_{i}\left(t\right)\in \left\{0,1\right\}$ is the task arrival indicator and $\mu_{i}\left(t\right)$ is the number of tasks served. The queuing delay $T_{i,\mathrm{ed}}^{q}$ is derived from the queue state as the difference between the execution start time and the task arrival time. Therefore, the total execution latency of the first *k* layers on the intelligent terminal is given by

(3) $$T_{i,ed}^{k,q}=T_{i,ed}^{k}+T_{i,ed}^{q},$$

where $T_{i,ed}^{k}$ is the pure computation latency of the first *k* layers on the ED, $T_{i,ed}^{q}$ is the queuing waiting latency on the ED. Since the types of DNN tasks executed on terminal devices are limited, trained DNN models can be pre-deployed and executed on UAVs. The computation latency of the remaining $L_{i}-k$ layers on the UAV, denoted as $T_{i,uav}^{k}$, is measured for each $k\in \{0,1,\ldots ,L_{i}\}$. This real-time measurement approach ensures that the latency data accurately reflect the actual execution environment on both the intelligent terminals and the UAVs, thereby providing a reliable basis for subsequent partitioning decisions and joint optimization.

### 3.3. Task Offloading

Intelligent terminals have limited local computational resources. Therefore, portions of DNN tasks are offloaded to UAVs through wireless networks. UAV $u_{m}$ is assumed to hover at a fixed altitude *h*. Its horizontal coordinates are denoted as $c_{uav}^{m}=\left(x_{uav}^{m},y_{uav}^{m}\right)$. The horizontal coordinates of intelligent terminal $v_{n}$ are given by $c_{ed}^{n}=\left(x_{ed}^{n},y_{ed}^{n}\right)$. The Euclidean distance between UAV $u_{m}$ and intelligent terminal $v_{n}$ is calculated as

(4) $$d_{n,m}=\sqrt{{\left(c_{uav}^{m}-c_{ed}^{n}\right)}^{2}+h^{2}},$$

where ∥·∥ denotes the Euclidean norm.

The altitude of the UAV enables Line-of-Sight (LoS) transmission to intelligent terminals within its visible range. Therefore, the wireless channel between UAV $u_{m}$ and intelligent terminal $v_{n}$ is assumed to be dominated by the LoS component. Following [17], the channel power gain is given as follows

(5) $$g_{n,m}=\beta_{0}\;d_{n,m}^{-2},$$

where $\beta_{0}$ represents the channel power gain at a reference distance of 1 m.

Orthogonal Frequency Division Multiple Access (OFDMA) is employed in SAGSIN. The channel is divided into smaller subchannels that are allocated to specific devices or device groups. This mechanism allows one access point to communicate simultaneously with multiple devices and effectively avoids interference among devices. The transmission rate for offloading tasks from intelligent terminal $v_{n}$ to UAV $u_{m}$ is given by

(6) $$R=b_{n,m}\log_{2}\left(1+\frac{g_{n,m}P_{n,m}}{b_{n,m}N_{0}}\right),$$

where $b_{n,m}$ is the bandwidth allocated by UAV $u_{m}$ to intelligent terminal $v_{n}$. $p_{n,m}$ is the transmission power from intelligent terminal $v_{n}$ to UAV $u_{m}$, $N_{0}$ is the noise power spectral density at the UAV.

The transmission latency for offloading the output data of the *k*-th layer of DNN task $j_{i}$ from the intelligent terminal to UAV $u_{m}$ is calculated as

(7) $$T_{i,\mathrm{tran}}^{k}=\frac{D_{i}^{k}}{R},$$

where $D_{i}^{k}$ denotes the data size of the *k*-th layer output that must be offloaded for task $j_{i}$.

The current system model assumes fixed-position hovering of UAVs during each service cycle. Negligible Doppler effect is observed under this assumption [27]. The proposed offloading model characterizes the fundamental trade-off between local computation, wireless transmission latency, and UAV-side execution latency. It lays the foundation for joint optimization of bandwidth allocation, task scheduling, and DNN partition points.

### 3.4. Problem Formulation

Based on the above analysis, this work investigates the joint optimization of three strategies in SAGSIN: bandwidth allocation strategy for intelligent terminals, task scheduling strategy on intelligent terminals, and DNN partitioning strategy based on task characteristics. The goal is to minimize the total processing latency of all DNN tasks. This problem is nonlinear and non-convex. The bandwidth allocation strategy is denoted as $b=\{b_{1},b_{2},\ldots ,b_{M}\}$, where *M* is the number of UAVs, and for each UAV *m*, $b_{m}=\left\{b_{m,1},b_{m,2},\ldots ,b_{m,N_{m}}\right\}$ with $N_{m}$ being the number of intelligent terminals connected to UAV *m*. The DNN model partitioning points are denoted as $k=\{k_{1},k_{2},\ldots ,k_{I}\}$, where *I* is the total number of DNN tasks, and $k_{i}$ is the partitioning point for task $j_{i}$. Channel power gains between UAVs and intelligent terminals are assumed to be known. Task priority calculation formulas are predetermined. The total processing latency minimization problem is formulated ams follows:

(8) $$\begin{array}{cc} \; & P:\;\min_{b,k}\sum_{i\in I}T_{i}^{\mathrm{tot}} \end{array}$$

(8a) $$\begin{array}{cc} \; & s.t.\;\sum_{i=1}^{N_{m}}b_{m,n}\leq B_{m},\;\forall m \end{array}$$

(8b) s.t.Nm≤αm

(8c) s.t.∑i=1Mαm≥N

(8d) s.t.Titot≤Timax

Constraint (8a) limits the total bandwidth allocated by UAV $u_{m}$ to its connected intelligent terminals, where $N_{m}$ is the number of terminals associated with UAV $u_{m}$. Constraint (8b) restricts the number of intelligent terminals that UAV $u_{m}$ can connect to, with $a_{m}$ being the maximum service capacity of UAV $u_{m}$. Constraint (8c) ensures sufficient UAV deployment to cover all intelligent terminals, where *N* is the total number of terminals and *M* is the total number of UAVs. Constraint (8d) guarantees that the total processing latency of each DNN task $j_{i}$ does not exceed its maximum tolerable latency $T_{i}^{\max}$. Task scheduling order is excluded from the DRL action space to avoid the combinatorial complexity of O(n!). It is determined by the weighted priority formula in Equation (16) with O(nlogn) complexity. The scheduling policy influences the objective indirectly through the queuing delay $T_{i,\mathrm{ed}}^{q}$ in $T_{i,\mathrm{tot}}^{k}$.

This formulation captures the coupling among bandwidth allocation, task scheduling, and DNN partitioning in a dynamic, resource-constrained UAV-assisted MEC environment. Due to its non-convex and nonlinear nature, traditional convex optimization techniques are difficult to apply effectively. Therefore, DRL is adopted in this work to seek near-optimal solutions.

## 4. Joint Model Segmentation and Resource Allocation Based on Deep Reinforcement Learning (DRL)

Flexibility, efficiency, and adaptability are offered by DRL technology. The constructed joint optimization problem is effectively solved by DRL. Traditional optimization methods rely on subproblem decomposition and convex relaxation. In contrast, DRL performs global optimization on the original non-convex problem through gradient descent and deep neural networks. High-dimensional variables are mapped to low-dimensional features by neural networks in DRL. Effective handling of high-dimensional variables is thereby achieved. Continuous interaction between the agent and the environment, combined with the adaptive decision-making mechanism, further enhances the capability of DRL to address inherently dynamic problems and obtain optimal long-term policies. Therefore, the formulated problem is solved by DRL methods in this paper. Optimal task scheduling strategies are obtained.

In this section, the joint optimization problem is first reformulated as a Markov Decision Process (MDP). Subsequently, generalized advantage estimation and proximal policy optimization are incorporated into the framework. A novel deep reinforcement learning-based algorithm for joint task scheduling and resource allocation is proposed. Finally, the computational complexity of the proposed algorithm is analyzed.

### 4.1. MDP Formulation

The Asynchronous A3C algorithm is a policy-gradient-based reinforcement learning method. Multiple parallel agents interact asynchronously with the environment in A3C. The policy and value function are approximated separately by the Actor and Critic networks. Fast and stable reinforcement learning training is thereby achieved. The A3C training framework includes one global neural network model and n worker threads. Each thread shares the same network structure as the global neural network. Each thread interacts independently with the environment. Experience data of a certain scale is collected. Losses for the Actor and Critic networks are computed independently according to Equations (10) and (11). Corresponding gradients are calculated. The computed gradients are asynchronously transmitted to the global neural network. Parameters of the global neural network are updated via gradient descent. The updated parameters are then synchronized to all threads. This process iterates repeatedly until model convergence is reached. Selected actions are evaluated in the algorithm through quantification of the advantage function.

The advantage function of the A3C algorithm is given by

(9) $$A\left(s_{t}\right)=r_{t}+\gamma r_{t+1}+\cdots +\gamma^{t+n-1}r_{t+n-1}+\gamma^{n}V\left(s_{t+n}\right)-V\left(s_{t}\right),$$

where $s_{t}$ is the state, $V(s_{t})$ is the state value function, γ∈[0,1] is the discount factor, and $r_{t}$ is the reward value.

The loss function of the Actor network is given by

(10) $$L_{\theta }=-\frac{1}{T}\sum_{t=1}^{T}\log\pi_{\theta }\left(a_{t}|s_{t}\right)\cdot A\left(s_{t}\right)-c\cdot H\left(\pi_{\theta }\left(a_{t}|s_{t}\right)\right),$$

where $\pi_{\theta }\left(a_{t}|s_{t}\right)$ is the policy function, $H(\pi_{\theta }\left(\cdot |s_{t}\right))$ is the entropy regularization term of policy $\pi_{\theta }$, and *c* is the weight of the entropy term. The entropy regularization term of policy $\pi_{\theta }\left(a_{t}|s_{t}\right)$ is added to prevent premature convergence to suboptimal policies.

The loss function of the Critic network is given by

(11) $$L_{\phi }=\frac{1}{T}\sum_{t=1}^{T}\delta^{2}\left(s_{t}\right),$$

where $\delta_{t}$ denotes the temporal difference error. In the A3C algorithm, the n-step temporal difference error takes the same mathematical form as the advantage function. The temporal difference error is defined as the difference between the Critic network of value prediction $V(s_{t})$ for state $s_{t}$ and the true target value of that state.

The PPO algorithm is a policy-gradient-based reinforcement learning method. The classic dual-network structure of Actor-Critic is inherited in PPO. Sample efficiency is effectively improved by introducing importance sampling and proximal policy optimization constraints. Training stability is enhanced. The core objective of the Critic network is to make the predicted value as close as possible to the true value target. The loss function is computed using Equation (13). Network parameters are updated via gradient descent. The core task of the Actor network is to increase the execution probability of advantageous actions. The agent is guided to select actions that yield high returns. The objective function is computed using Equation (15). Network parameters are updated via gradient ascent. A clipping constraint is added to the objective function in the Actor network to prevent abrupt policy changes. Training stability is ensured. Moreover, the objective function depends only on the action probability ratio between the old policy during sampling and the current new policy. The objective function is independent of subsequent policy changes. The old policy is fixed. The same batch of data can be reused for multiple updates. Sample utilization is thereby improved. Sampling computational cost is reduced.

The true value objective formula for the PPO algorithm is

(12) $$V_{t}^{\mathrm{target}}=r_{t}+\gamma \cdot V_{\phi }\left(s_{t+1}\right),$$

where $r_{t}$ is the immediate reward received by the agent after executing action $a_{t}$, γ is the discount factor, and $V_{\phi }\left(s_{t+1}\right)$ is the predicted value of state $s_{t+1}$.

The loss function of the Critic network is given by

(13) $$L_{\phi }=E_{t}\left[{\left(V_{\phi }\left(s_{t}\right)-V_{t}^{\mathrm{target}}\right)}^{2}\right].$$

The advantage function is given by

(14) $$A_{t}=V_{t}^{\mathrm{target}}-V_{\phi }\left(s_{t}\right),$$

where $V_{t}^{\mathrm{target}}$ is the true value target of state $s_{t}$, and $V_{\phi }\left(s_{t}\right)$ is the predicted value of state $s_{t}$.

The objective of the Actor network is to maximize the following objective function:

(15) $$J_{\theta }^{\mathrm{clip}}=E_{t}\left[\min\left(r_{t}\left(\theta \right)A_{t},\mathrm{clip}\left(r_{t}\left(\theta \right),1-\epsilon ,1+\epsilon \right)A_{t}\right)\right],$$

where $r_{t}\left(\theta \right)=\frac{\pi_{\theta }\left(a_{t}|s_{t}\right)}{\pi_{\theta^{\mathrm{old}}}\left(a_{t}|s_{t}\right)}$ is the probability ratio between the new and old policies, $\pi_{\theta }\left(a_{t}|s_{t}\right)$ is the probability of action $a_{t}$ under the new policy, and $\pi_{\theta^{\mathrm{old}}}\left(a_{t}|s_{t}\right)$ is the probability under the old policy. When $r_{t}\left(\theta \right)>1$, the new policy assigns higher probability to the action than the old policy. The clipping constraint limits $r_{t}\left(\theta \right)$ to the interval [1−ϵ,1+ϵ] to ensure stable policy iteration.

### 4.2. Scheduling Algorithm Based on Computation Delay Weighted Remaining Time Priority

Bandwidth allocation strategies and task offloading strategies for intelligent terminals are formulated by the UAV as an agent. These strategies are based on the total available bandwidth, the number of connected intelligent terminals, and the structure of pending DNN models. Decisions are executed by the agent. Integration of a domain-specific scheduling mechanism into the deep reinforcement learning loop is implemented. The weighted priority scheduling algorithm is embedded within the reward computation pipeline of the DRL framework, rather than functioning as an independent external module. The queuing delay $T_{i,\mathrm{ed}}^{q}$ is directly influenced by this embedded mechanism. The optimization landscape of both A3C and PPO is shaped by the modified delay metric. The bandwidth allocation and task offloading strategies are updated according to the received rewards.

Continuous task arrivals are considered. Execution of multiple tasks on the intelligent terminal requires priority-based queuing. A non-preemptive task scheduling algorithm based on computation latency-weighted remaining time priority is adopted in this paper.

Task arrivals are assumed to be random. The starting time of task $j_{i}$ is denoted as $t_{i}^{\mathrm{ini}}$. The priority of task $j_{i}$ at time *t* is set as

(16) $$P_{i}\left(t\right)=\alpha \cdot \left(1-\frac{T_{i}^{\mathrm{rem}}}{T_{\max}^{\mathrm{rem}}}\right)+\beta \cdot \left(1-\frac{T_{i,\mathrm{ed}}^{k}}{T_{\max,\mathrm{ed}}^{k}}\right)+\gamma \cdot \tanh\left(\lambda \cdot \frac{T_{i}^{\mathrm{wait}}}{T_{\max}^{\mathrm{wait}}}\right),$$

where $T_{i}^{\mathrm{rem}}=T_{i}^{\max}+t_{i}^{\mathrm{ini}}-t$ is the remaining available time of DNN task $j_{i}$ at time *t*, $T_{i}^{\mathrm{rem}}$ is the maximum tolerable total processing latency of DNN task $j_{i}$, and $T_{\max}^{\mathrm{rem}}$ is the maximum remaining time among all tasks currently running on the intelligent terminal. The computation latency of task $j_{i}$ on the intelligent terminal is denoted as $T_{i,\mathrm{ed}}^{k}$, while $T_{\max,\mathrm{ed}}^{k}$ is the maximum computation latency among all tasks currently executed on the intelligent terminal. The waiting time of task $j_{i}$ is denoted as $T_{i}^{\mathrm{wait}}$, and $T_{\max}^{\mathrm{wait}}$ is the maximum waiting time among all tasks currently running on the intelligent terminal. The coefficients α, β, and γ are weighting factors satisfying α+β+γ=1. The hyperbolic tangent (tanh) function is incorporated into the priority formula so that nonlinear constraints are imposed on the contribution of waiting time. Prolonged waiting of any single task is thus prevented from excessively dominating the global scheduling priority in dynamic edge environments of industrial Internet and wireless communication systems.

$P_{i}\left(t\right)$ is the computation latency-weighted remaining time priority of task $j_{i}$ at time *t*. Higher values indicate higher scheduling priority. The design of this priority formula integrates three factors: urgency of task remaining time, short-job-first principle, and avoidance of long-job starvation. Normalization mechanisms are introduced simultaneously. Weighted fusion of indicators across uniform dimensions is ensured. Tighter remaining available time leads to higher priority. Real-time requirements of tasks are thereby guaranteed. Shorter task computation latency leads to higher priority. Long-duration tasks are prevented from monopolizing resources and causing system blockage. Longer task waiting time leads to higher priority. Starvation of long jobs due to sustained waiting is prevented. Task priorities are dynamic. Priorities change with time. Therefore, priorities of all tasks are recalculated whenever a new task arrives. The new task is inserted into the waiting queue according to its priority. Non-preemptive scheduling is adopted in this algorithm. Computational resources are allocated to the task at the head of the waiting queue only when the intelligent terminal completes a task and becomes idle. The task is then removed from the waiting queue. The execution latency of the task on the intelligent terminal is given by $T_{i,\mathrm{ed}}^{k,q}=T_{i,\mathrm{ed}}^{k}+T_{i,\mathrm{ed}}^{q}$, where $T_{i,\mathrm{ed}}^{k}$ is the computation latency of task $j_{i}$ on the intelligent terminal, and $T_{i,\mathrm{ed}}^{q}$ is the queuing waiting latency of task $j_{i}$ on the intelligent terminal.

$T_{i,\mathrm{ed}}^{q}$ is defined as the starting execution time $t_{i,\mathrm{ed}}^{\mathrm{start}}$ on the intelligent terminal minus the starting time $t_{i}^{\mathrm{ini}}$ of task $j_{i}$. The task scheduling algorithm aims to minimize the total processing latency of all tasks on the intelligent terminal $\sum T_{i}^{\mathrm{tot}}$. All tasks are ensured to complete within their maximum tolerable total processing latency $T_{i}^{\max}$, i.e., $T_{i}^{\mathrm{tot}}\leq T_{i}^{\max}$.

### 4.3. Joint Optimization Method for Bandwidth Allocation and DNN Slicing Based on Weighted Priority Scheduling and A3C-PPO

**Figure 2:**
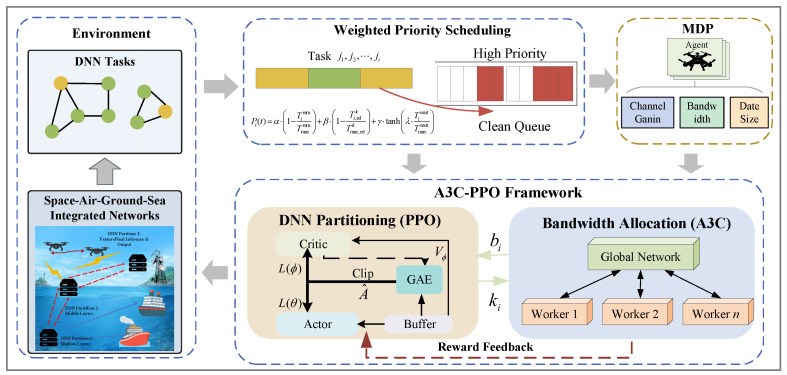
Framework of the Weighted Priority Scheduling and A3C-PPO algorithm.

The UAV cluster serves as the decision-making agent in the joint optimization framework for bandwidth allocation and DNN model partitioning based on the A3C-PPO hybrid algorithm. Bandwidth allocation strategies for each intelligent terminal are generated by the A3C algorithm. A tightly coupled closed-loop feedback mechanism is implemented. A loose connection between the two modules is avoided. Interaction is achieved through a bidirectional state feedback loop. The transmission rate *R* is determined by the output of A3C for bandwidth allocation. The transmission time $T_{i,\mathrm{tran}}^{k}=D_{i}^{k}/R$ is incorporated into the state space of PPO. Partitioning decisions are influenced by this metric. The offloaded data size $D_{i}^{k}$ is determined by the partitioning decision of PPO. This size is fed back into the state of A3C. The offloading demand is represented by this component. A co-evolutionary optimization dynamic is created by the bidirectional coupling. Such dynamic is absent in standard parallel or hierarchical architectures. Optimal offloading partitioning points for each DNN task are determined by the PPO algorithm. These strategies are based on the total available bandwidth of the agent, the current number of connected intelligent terminals, and the structure of pending DNN models. A collaborative decision scheme of “bandwidth allocation-task partitioning offloading” is formed. Rewards generated from environment interactions are received by the agent through a closed-loop feedback mechanism after decision execution. Network parameters of the dual strategies are iteratively optimized based on the rewards. Continuous improvement of the overall system performance is ultimately achieved. The algorithm framework is illustrated in Figure 2.

The system dynamically allocates uplink bandwidth to each connected intelligent terminal using the A3C algorithm. The distributed “global policy network + multiple worker agents” architecture offers significant advantages in UAV-assisted MEC networks: multiple worker agents enable parallel sampling, substantially accelerating policy convergence, while the global policy network performs centralized state evaluation to avoid fragmented bandwidth allocation and achieve optimal global resource configuration. During deployment, one UAV serves as the central node hosting the global policy network for coordinated decision-making, while the remaining UAVs act as worker agents responsible for local state collection and policy sampling. The state space, action space, and reward function are designed as follows:

State space: Data transmission demands of intelligent terminals, bandwidth resource constraints of UAVs, and the network environment are comprehensively considered. The input state includes channel gains, the number of terminals connected to each UAV, task partitioning, offloading data sizes, and the available bandwidth of UAVs. The state space is defined as $S_{A3C}=\left\{\left\{g_{1},g_{2},\cdots ,g_{M}\right\},\left\{N_{1},N_{2},\cdots ,N_{M}\right\},\left\{B_{1},B_{2},\cdots ,B_{M}\right\},\left\{D_{1},D_{2},\cdots ,D_{M}\right\}\right\}$, where $g_{m}=\left\{g_{m,1},g_{m,2},\cdots ,g_{m,N_{m}}\right\}$, and $g_{m,i}$ denotes the channel gain between intelligent terminal $v_{n}$ and UAV $u_{m}$; $N_{m}$ is the number of intelligent terminals currently connected to UAV $u_{m}$; $B_{m}$ is the current available bandwidth of UAV $u_{m}$. $D_{m}=\left\{D_{m,1}^{k_{1}},D_{m,2}^{k_{2}},\cdots ,D_{m,N_{m}}^{k_{N_{m}}}\right\}$, and $D_{m,i}^{k_{i}}$ is the data size that needs to be offloaded to UAV $u_{m}$ for the task running on intelligent terminal $v_{n}$ at partitioning point $k_{n}$.

Action space: The action space of A3C is set as the bandwidth selection for intelligent terminals connected to UAVs. The action space is denoted as $A_{A3C}=\left\{b_{1},b_{2},\ldots ,b_{M}\right\}$, where $b_{m}=\left\{b_{m,1},b_{m,2},\cdots ,b_{m,N_{m}}\right\}$, and $b_{m,i}$ is the proportion of available bandwidth allocated by UAV $u_{m}$ to intelligent terminal $v_{n}$, with $\sum_{i=1}^{N_{m}}b_{m,i}=1$, and $N_{m}$ is the number of intelligent terminals connected to UAV $u_{m}$.

Reward function: The reward function guides A3C to select the optimal bandwidth allocation strategy. The average transmission latency of all terminals is minimized. The reward function is defined as

(17) $$R_{A3C}=-{\bar{T}}_{\mathrm{tran}},$$

where ${\bar{T}}_{\mathrm{tran}}$ is the average transmission latency of all terminals. Transmission latency is reduced through negative penalties.

Intelligent decisions for DNN model partitioning points are implemented by the system using the PPO algorithm. The decision module is deployed on the UAV side. The optimization objective of this decision is to minimize total task processing latency and maximize task completion rate. Collaborative feedback is formed with the bandwidth allocation strategy of A3C. Overall system performance is enhanced. Strong training stability is possessed by the PPO algorithm. Parameter oscillations caused by reward fluctuations and action space changes during training are effectively avoided by its clipping mechanism. Scenarios with diverse terminal tasks and dynamic load changes are adapted. The state space, action space, and reward function of the PPO algorithm are designed as follows:

State space: Characteristics of DNN tasks and feedback information from A3C bandwidth allocation are comprehensively considered. The input state includes execution latencies of DNN tasks on intelligent terminals and UAVs, and transmission latency from intelligent terminals to UAVs. The state space is denoted as $S_{PPO}=\left[T_{i,\mathrm{ed}}^{k},T_{i,\mathrm{tran}}^{k},T_{i,\mathrm{uav}}^{k}\right]$, where $T_{i,\mathrm{ed}}^{k}$ is the execution latency of the first $k_{i}$ layers of task $j_{i}$ on the intelligent terminal. $T_{i,\mathrm{tran}}^{k}$ is the transmission latency of the $k_{i}$-th layer data from the intelligent terminal to the UAV for task $j_{i}$, $T_{i,\mathrm{uav}}^{k}$ is the execution latency of the remaining $L_{i}-k_{i}$ layers of task $j_{i}$ on the UAV.

Action space: The action space of PPO is discrete. Each DNN task of partitioning point index decision is corresponded. The action space is denoted as $A_{PPO}=\left\{k_{i}\right\}$, where $k_{i}$ is the partitioning point of DNN task $j_{i}$. The first $k_{i}$ layers of task $j_{i}$ are executed locally on the intelligent terminal. The remaining $L_{i}-k_{i}$ layers are offloaded to the UAV for execution.

Reward function: The reward function guides PPO to select the optimal DNN task partitioning point. Total task processing latency is minimized. Task completion rate is maximized. The reward function is defined as

(18) $$R_{PPO}=-{\bar{T}}_{\mathrm{tot}},$$

where $T_{\mathrm{tot}}=T_{\mathrm{ed}}+T_{\mathrm{tran}}+T_{\mathrm{uav}}$ is the average total processing latency of all tasks.

The A3C and PPO modules interact through a tightly coupled closed-loop feedback mechanism. Specifically, A3C of bandwidth allocation output $b_{n,m}$ directly determines the transmission rate *R* via Equation (6), which enters PPO of state space as the transmission latency $T_{i,\mathrm{tran}}^{k}=D_{i}^{k}/R$, thereby influencing the optimal partitioning point. Conversely, PPO of partitioning decision $k_{i}$ determines the offloaded data size $D_{i}^{k}$ from the DNN model structure, which feeds back into A3C of state as a key demand-side input $D_{m}$. This bidirectional coupling ensures that the two modules co-evolve toward a globally coordinated optimum rather than converging to independently optimal but jointly suboptimal solutions.

### 4.4. Algorithm Design

The training of the A3C-PPO framework follows a sequential three-stage procedure. In Stage 1, the PPO module is pre-trained offline with an equal-share bandwidth baseline to learn a near-optimal DNN partitioning policy. In Stage 2, the A3C module is trained with the PPO policy frozen, optimizing bandwidth allocation based on the offloading demands determined by the PPO of fixed partitioning decisions. In Stage 3, an optional fine-tuning phase allows limited joint updates with reduced learning rates to capture residual coupling effects. During deployment, both policies operate in inference mode: PPO first determines the partitioning point, and A3C subsequently adjusts bandwidth allocation, ensuring real-time and deterministic decision-making on UAV edge nodes. The training process of the algorithm is given in Algorithm 1 and is explained as follows.
**Algorithm 1** Training of the Joint Optimization Strategy for Bandwidth Allocation and DNN Task Partitioning Based on A3C-PPO (Worker Side)**Require:** UAV Info, UE Info, Link Info, DNN task**Ensure:** Joint optimization policy for bandwidth allocation and DNN task partitioning at smart terminals  1:Initialize system state; synchronize worker neural network parameters with global network  2:**for** episode = 1 **to** max_episodes **do**  3:   **for** sub_episode = 1 **to** max_sub_episodes **do**  4:      Given current state $S_{t}$, select action $A(S_{t})$ using worker’s policy network to obtain bandwidth allocation strategy  5:      Execute action $A(S_{t})$; apply trained PPO policy to determine optimal DNN partitioning point *k*  6:      Obtain next state $S_{t+1}$ and reward $r_{t}$; set $S_{t+1}$ as input for next step  7:      Normalize reward $r_{t}$ to obtain $n_{r_{t}}$  8:      Store transition $\left(S_{t},A\left(S_{t}\right),n_{r_{t}}\right)$ in local buffer  9:      **if** sub_episode mod 3 = 0 **or** sub_episode = max_sub_episodes **then**10:          {Parameter update condition}11:          Compute state value $V(S_{t})$ using stored transitions12:          Calculate actor and critic losses; compute gradients $\nabla_{\theta }L_{\theta }$, $\nabla_{\phi }L_{\phi }$13:          Asynchronously push gradients to global network; reset local buffer14:     **end if**15:   **end for**16:**end for**

The PPO algorithm ensures stability in policy optimization. By employing truncated surrogate objective functions, it avoids drastic policy updates. This guarantees monotonic performance improvement throughout training. The A3C architecture effectively reduces sample correlation. Multiple parallel workers handle environmental interactions. Asynchronous updates enhance data diversity. Consequently, the A3C-PPO framework achieves robust convergence. The proposed system balances training stability with sample efficiency.

## 5. Simulation Configuration

Key parameter settings of the UAV-assisted MEC network are presented in this section. Hyperparameter configurations adopted by the A3C algorithm, the PPO algorithm, and the weighted priority scheduling algorithm are also described. Experimental results are then analyzed. Conclusions are drawn from the analysis. The overall system performance improvement achieved by the proposed joint optimization method for bandwidth allocation and DNN partitioning based on weighted priority scheduling and A3C-PPO is thereby validated.

### 5.1. Simulation Parameter Settings

Three UAVs are deployed in the UAV-assisted edge network under SAGSIN scenarios. UAVs hover at heights of 40–100 m above ground to execute predefined tasks. The network coverage area is 500 m × 500 m. Intelligent terminals are randomly distributed within the coverage area. Four intelligent terminals are directly connected to each UAV. The uplink transmission power of each intelligent terminal is set to 26 dBm. The noise power spectral density $N_{0}$ between UAVs is −170 dBm/Hz. The channel power gain $\beta_{0}$ at a 1-m distance is −20 dB. DNN inference tasks are generated continuously at random intervals. Specific parameter settings are listed in Table 2.

Core parameter configurations of the A3C-PPO-based joint optimization strategy are presented in Table 2. The state space of the MDP formulation comprises states of UAVs, information of intelligent terminals, qualities of communication links, and structural features of DNN models. The action space is denoted as a joint strategy of bandwidth allocation ratios for terminals and partitioning layers for DNN models. The clipping factor of PPO, denoted as ϵ, is set to 0.2. The magnitude of policy updates is constrained by this factor. Training stability is thereby ensured. The generalized advantage estimation parameter, denoted as λ, is set to 0.95. A balance between estimation bias and variance is achieved. Three comparative values for the learning rate are given as $1\times 10^{-5}$, $5\times 10^{-6}$, and $1\times 10^{-6}$. Impacts on convergence performance are analyzed through these configurations. The parameter synchronization interval between worker networks and the global network is set to 3. Parameter synchronization is performed every 3 sub-episodes. Robust convergence of the training process is ensured by this mechanism.

### 5.2. Algorithm Performance Analysis

Multiple neuron layers with identical functions in the DNN model are defined as one layer. The entire DNN model is partitioned into two parts layer by layer. The front part is computed by the intelligent terminal. The rear part is offloaded to the UAV via wireless links. The rear part is computed by the MEC server on the UAV. Experimental results demonstrate the existence of an optimal partitioning point. The total execution latency of the entire model is minimized at this point. The total available bandwidth *w* of UAV *u* is 20 MHz. The bandwidth allocated to intelligent terminal *v* through the bandwidth allocation strategy is 6.668743 MHz. The data transmission rate is 84.5 Mbps. Execution latencies vary across different model partitioning points for the ResNet18 model task. The numbers on the x-axis represent the partitioning positions of the model. Position 1 indicates that the partitioning is at the input layer. Input data serves as the transmission data. The entire model’s computation task is undertaken by the MEC server on the UAV. Position 9 indicates that the partitioning is at the end. No offloading is performed. The entire model is computed by the intelligent terminal itself. The y-axis represents task execution latency. The unit is seconds. The DNN model parts executed by the UAV and intelligent terminal, along with the data sizes to be transmitted, at different partitioning positions of ResNet18 are listed in Table 3.

Note: ResNet18 includes one convolutional layer Conv1 (Conv + Batch Normalization + ReLU + MaxPool) + 4 residual layers Conv2–Conv5 (each residual layer has 2 residual blocks, totaling 8 residual blocks), and one fully connected layer (AvgPool + FC Layer). Table 3 compares the computational overhead of different partitioning decision methods. For ResNet18 with 9 partitioning positions, exhaustive search requires $O(L_{i})$ evaluations per task, while PPO inference requires only a single neural network forward pass with O(1) complexity relative to the number of layers. This constant-time decision-making is particularly advantageous for deeper DNN models and for real-time deployment on resource-constrained UAV platforms, where decision latency directly impacts the overall task processing time.

The computation speed of the server deployed on the UAV typically far exceeds that of the intelligent terminal. From a theoretical perspective, as many DNN layers as possible are preferred to be offloaded to the UAV for computation. However, task execution latency includes computation latency of the intelligent terminal, computation latency of the UAV server, and data transmission latency from the intelligent terminal to the UAV. Therefore, data sizes to be transmitted at different layers must be considered when determining the partitioning point of DNN tasks. For the ResNet18 model, the data size to be transmitted at the 2nd layer is as high as 2,097,152 bits. In contrast, the 8th layer requires only 16,384 bits. Therefore, the sum of computation latencies for the intelligent terminal and UAV server is much smaller when the partitioning point is set at the 2nd layer, as the UAV undertakes more computation tasks of the DNN task. However, the transmission time at this point is much larger than at the 8th layer due to the large data size. In this case, the total task execution latency increases significantly because of the notable rise in transmission time.

**Figure 3:**
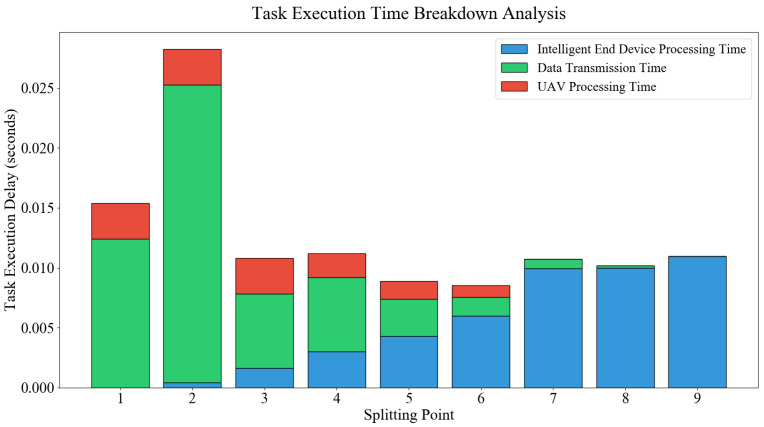
Execution Latency of ResNet18 under Different Partitioning Points with a Data Transmission Rate of 84.5 Mbps.

**Figure 4:**
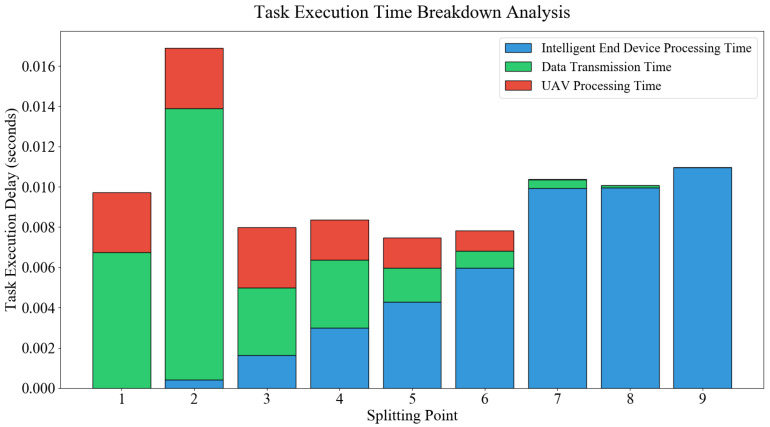
Execution Latency of ResNet18 under Different Partitioning Points with a Data Transmission Rate of 155.6 Mbps.

The optimal partitioning position for the ResNet18 model is the 6th layer when the total available bandwidth of the UAV is 20 MHz, as shown in Figure 3. The task execution latency $T_{n,\mathrm{tot}}=T_{n,\mathrm{ed}}+T_{n,\mathrm{tran}}+T_{n,\mathrm{uav}}$ is minimized. Figure 4 shows the execution latencies at different partitioning points for the ResNet18 model task when the total available bandwidth *w* of UAV *u* is 40 MHz, the bandwidth allocated to intelligent terminal *v* is 13.330940 MHz, and the data transmission rate is 155.6 Mbps. The optimal partitioning position for the ResNet18 model is the 5th layer when the total available bandwidth is 40 MHz, as shown in Figure 4. The data transmission speed increases significantly when the allocated bandwidth for the intelligent terminal rises from 6.668743 MHz to 13.330940 MHz. Transmission time is thereby shortened. The influence of transmission time on total latency is relatively reduced. Consequently, the partitioning point shifts from the 6th layer to the 5th layer. Bandwidth affects the DNN model partitioning position, as demonstrated by this.

**Figure 5:**
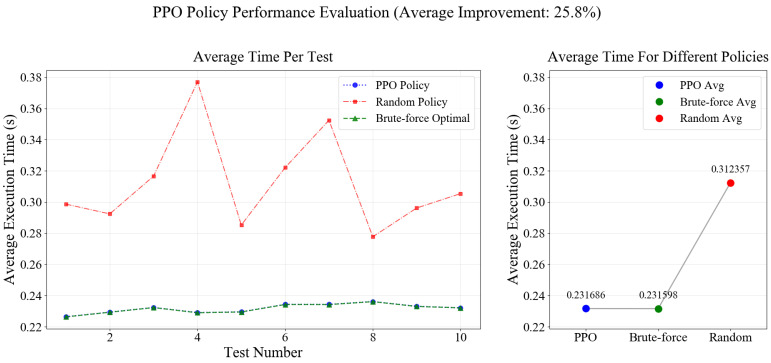
PPO Strategy Performance Analysis.

A joint optimization method based on A3C-PPO for bandwidth allocation and DNN partitioning is proposed in this chapter. This chapter proposes a joint optimization method for bandwidth allocation and DNN partitioning based on A3C-PPO. To evaluate the performance of the PPO strategy in DNN task partitioning, the following experimental environment was designed. The parameters of the PPO strategy algorithm are as follows: discount factor γ=0.9, GAE parameter λ=0.95, clip factor ε=0.2, PPO update epochs = 8, batch size =32, learning rate $1\times 10^{-4}$, entropy coefficient 0.02, and value function coefficient 0.5. The reward function is defined as the negative value of the normalized total task latency. The total available bandwidth for the UAV varies randomly within the range of 20–60 MHz, and the bandwidth value for each task is generated independently to simulate a dynamic environment. ResNet18 is adopted as the main DNN inference task model. Based on the model structure, it is divided into 9 logical layers. The local computation time, remote computation time, and transmission data volume corresponding to each partition point are obtained based on actual measurements. Among these, local computation time is executed by the intelligent terminal, remote computation time is executed by the UAV MEC server, and the transmission data volume corresponds to the size of the intermediate feature maps. After training is completed, 10 rounds of tests are randomly generated (1000 tasks per round). The PPO strategy, random partitioning strategy, and brute-force search optimal partitioning strategy are respectively adopted to calculate the total execution latency. The performance of the PPO strategy is shown in Figure 5. The left figure shows the performance of the PPO algorithm and its comparison algorithms over 10 rounds of tests. The y-axis represents the average of the sum of DNN inference task latencies for a specific test round (in seconds), and the x-axis represents the number of test rounds. The right figure compares the total average values of the PPO algorithm and its comparison algorithms after 10 rounds. As can be seen from the figure, DNN tasks are partitioned using three methods: random partitioning, brute-force search for the optimal partition point, and the proposed PPO strategy. The average task execution time under the random strategy is 0.312357 s, the average task execution time for the brute-force search for the optimal partition is 0.231598 s, and the average task execution time under the PPO strategy is 0.231686 s. Compared with the random partitioning strategy, the performance is improved by 25.83%. Compared with the brute-force search for the optimum, the gap is only 0.04%. This indicates that the proposed PPO strategy can accurately find the DNN task partition points.

**Figure 6:**
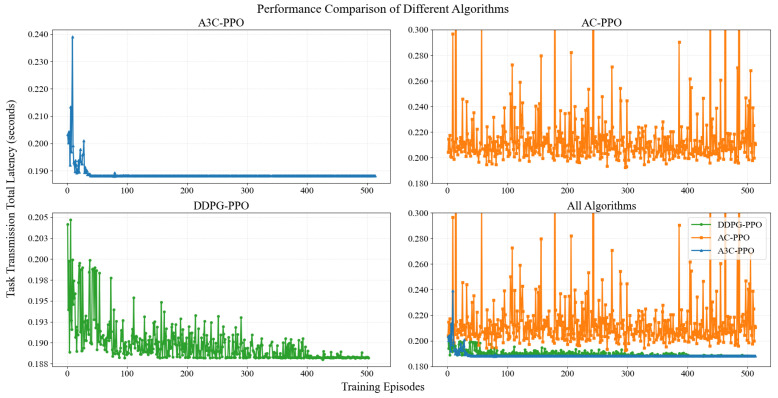
Performance Comparison of A3C-PPO, AC-PPO, and DDPG-PPO Algorithms.

The A3C network is analyzed next. The A3C algorithm is compared with the AC algorithm and DDPG, as shown in Figure 6. The y-axis represents the sum of DNN inference task latencies. The unit is seconds. Figure 6 illustrates a DNN task based on ResNet18, which consists of an initial convolutional layer, four residual block groups, totaling eight residual blocks, and a fully connected layer. To facilitate partitioning experiments, the network is decomposed into nine logical layers composed of consecutive neural layers, as detailed in Table 3. With an input data volume of 1,048,576 bits (1 MB), the size of the intermediate feature maps requiring transmission varies significantly depending on the partitioning point. This variation directly dictates the trade-off between transmission latency and computational latency. Asynchronous training is achieved by A3C through parallelization of multiple worker threads or processes. Each thread interacts with the environment in different states. Parameters of the global network are updated asynchronously by each thread. Convergence speed of the algorithm is significantly improved by this parallelization. Diverse sample data is generated independently by each thread. Exploration capability in the state space is further enhanced by sample diversity. Data utilization efficiency is thereby increased. Consequently, A3C exhibits clear advantages over AC and DDPG in asynchronous training, sample diversity, data utilization efficiency, and algorithm stability. The proposed A3C-PPO-based bandwidth allocation algorithm converges to the optimal solution at the 41st episode, as shown in Figure 6. The fastest convergence speed and the most stable performance are demonstrated compared with AC and DDPG.

The AC algorithm fails to converge under the same conditions, as shown in Figure 6. The AC algorithm employs only a single worker for training. Policy gradient variance is high. Sample correlation is strong. The advantage of asynchronous training cannot be utilized to break sample correlation. Variance during training cannot be effectively reduced. Actor and Critic are updated simultaneously. The value function of Critic changes continuously with the policy. A moving target problem is formed. Training oscillation is thereby caused. Stability and convergence of AC are clearly inferior to those of A3C. The DDPG algorithm tends to stabilize around the 400th episode, as shown in Figure 6. Convergence speed of DDPG is significantly slower than that of A3C.

The effectiveness of the proposed A3C-PPO hybrid architecture is verified through comparative experiments. Two baseline strategies are constructed for evaluation. The bandwidth allocation strategy based on A3C is denoted as Only-A3C. The model partitioning strategy based on PPO is denoted as Only-PPO. Comparisons are conducted between the baseline strategies and the A3C-PPO joint optimization method.

**Figure 7:**
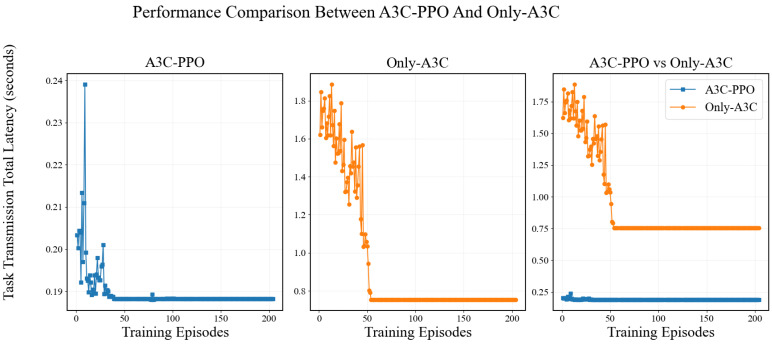
Performance Comparison of A3C-PPO and Only-A3C Algorithms.

The Only-A3C strategy employs the A3C algorithm for bandwidth allocation exclusively. Model partitioning optimization via PPO is not incorporated. Due to the absence of a model partitioning mechanism, the complete computational workload of all DNN tasks is offloaded to the UAV-mounted server for execution. The experimental environment is configured identically to that of the A3C-PPO framework. Four intelligent terminals are connected to the UAV. Inference tasks are continuously generated by each terminal. The performance comparison between Only-A3C and A3C-PPO is presented in Figure 7. Convergence is achieved by the Only-A3C strategy. However, the converged objective value of Only-A3C is significantly higher than that of A3C-PPO. This performance gap is attributed to the full offloading of computational tasks to the UAV in Only-A3C. The volume of transmitted data is substantially increased. Consequently, transmission latency becomes the performance bottleneck. In contrast, a balance between transmission latency and computation latency is achieved by the A3C-PPO framework through joint optimization of bandwidth allocation and model partitioning. Superior overall latency performance is thereby obtained.

Model partitioning optimization is performed by the Only-PPO strategy via the PPO algorithm exclusively. Allocation of bandwidth resources is not conducted. Near-optimal performance is achieved by the Only-PPO strategy in the single-UAV-single-terminal scenario. The optimal partitioning point is identified based on a fixed transmission rate. However, bandwidth resources must be allocated among multiple terminals in the multi-terminal scenario. Dynamic adjustment of bandwidth is not supported by the Only-PPO strategy. Consequently, the transmission rate of each terminal remains uncertain.

**Figure 8:**
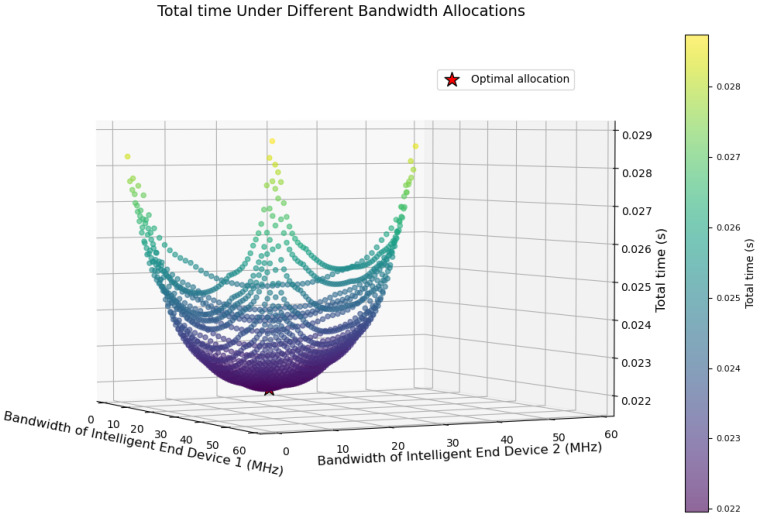
Variation of Total Task Execution Time versus Bandwidth Allocation under the Only-PPO Strategy.

An experiment is designed to evaluate the performance of the Only-PPO strategy in the multi-terminal environment. A single UAV is connected to three intelligent terminals. The distances between the UAV and the terminals are given as 500 m, 300 m, and 50 m, respectively. The total available bandwidth of the UAV is denoted as 60 MHz. DNN inference tasks are required to be completed by each terminal. Due to the absence of a bandwidth allocation strategy, bandwidth is allocated to each terminal via random sampling. The bandwidth sum constraint is satisfied during the allocation process. The optimal partitioning point for each terminal is independently determined by the PPO algorithm. The total completion latency of all tasks is calculated for evaluation. Multiple random sampling trials are conducted. The distribution of total latency versus bandwidth allocation is obtained. The results are presented in Figure 8. In Figure 8, each data point represents the total latency corresponding to a random bandwidth allocation instance. The color intensity of each point indicates the magnitude of the latency. A distinct optimal bandwidth allocation region is observed in Figure 8, which is indicated by the dark-colored area. The minimum total latency is achieved within this region. However, the capability of dynamic bandwidth allocation is not supported by the Only-PPO strategy. An active search for the optimal allocation region cannot be performed. Dependence on random sampling is inevitable. Consequently, the average total latency of Only-PPO is significantly higher than the optimal value achieved by the A3C-PPO framework. In contrast, bandwidth allocation and model partitioning strategies are simultaneously learned by the A3C-PPO framework through joint optimization. The optimal region presented in Figure 7 is thereby approached.

The limitations of single-algorithm dependencies are demonstrated by the comparative experiments. The full performance potential of the system cannot be exploited by either the Only-A3C or the Only-PPO strategy. Optimization of bandwidth allocation is achieved by the Only-A3C strategy. However, the transmission-computation trade-off induced by model partitioning is neglected. Accurate model partitioning is performed by the Only-PPO strategy. However, bandwidth competition among multiple terminals cannot be addressed. Collaborative optimization of both strategies is achieved by the A3C-PPO framework. The asynchronous parallel architecture and the proximal policy optimization mechanism are utilized. Task execution efficiency is significantly improved. The necessity and superiority of the hybrid architecture are thereby verified.

**Figure 9:**
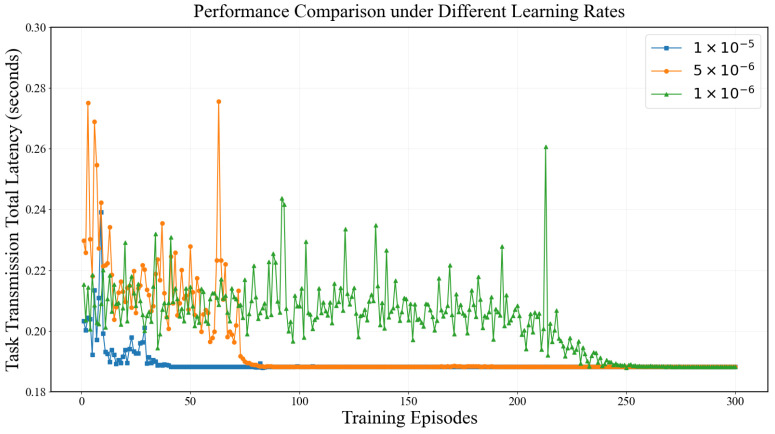
Comparison of A3C Algorithm Performance at Different Learning Rates.

Learning rate is a critical hyperparameter in the A3C algorithm. The step size of parameter updates during optimization is controlled by the learning rate. Different learning rate settings significantly affect the performance of A3C. The performance of A3C is compared at learning rates of $1\times 10^{-5}$, $5\times 10^{-6}$, and $1\times 10^{-6}$, as shown in Figure 9. Convergence speed is fastest when the learning rate is $1\times 10^{-5}$. Convergence speed gradually decreases from $1\times 10^{-5}$, to $1\times 10^{-6}$. Learning rates higher than $1\times 10^{-5}$, may cause oscillation near the optimal solution. Stable convergence to the final state may even become impossible. Learning rates that are too low make the convergence process slow. Significantly more iterations are required to reach the desired performance level. Reasonable selection of learning rate is essential to ensure efficiency and stability of the algorithm.

**Figure 10:**
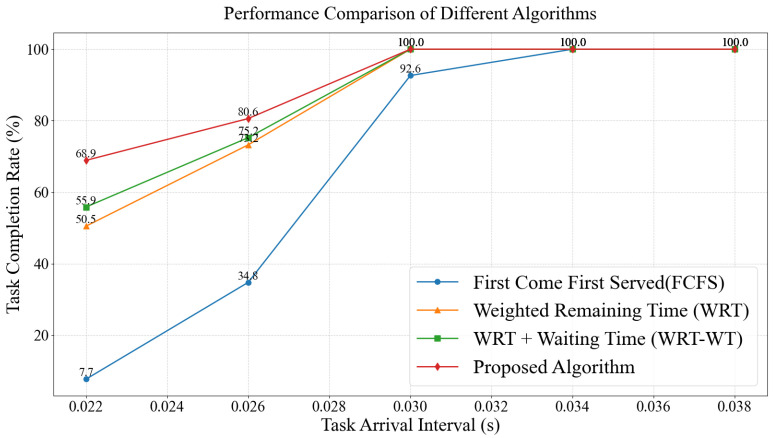
Task Completion Rate Comparison Between Weighted Least Remaining Time Priority Scheduling Algorithm and First-Come-First-Served Task Scheduling Algorithm.

Continuous task arrivals are considered. Execution of multiple tasks on the intelligent terminal requires priority-based queuing. Task completion rate is affected by different task scheduling algorithms. A priority scheduling algorithm based on computation latency-weighted remaining time and waiting time is introduced in this chapter. Task execution efficiency is optimized by this algorithm. Performance comparison of four algorithms is shown in Figure 10: First Come First Served (FCFS), Weighted Remaining Time (WRT), Weighted Remaining Time and Waiting Time (WRT-WT), and the proposed algorithm. DNN tasks are classified into urgent and non-urgent categories based on their maximum tolerable latency. Continuous generation of multiple DNN task types is simulated. Urgent tasks appear randomly. Fixed task arrival intervals are set to ensure a clear comparison. All four algorithms achieve 100% task completion rate when task arrival intervals are greater than or equal to 34 ms. Performance is comparable under light task load. The completion rate of FCFS drops by 7.4 percentage points when the interval decreases to 3 ms. All algorithms fail to complete all tasks under high-load conditions with intervals below 26 ms. The proposed algorithm still achieves a task completion rate of 80.6%. Superior performance under extreme conditions is demonstrated. The priority scheduling algorithm based on computation latency-weighted remaining time and waiting time excels in improving task execution efficiency on intelligent terminals. The advantage is more pronounced in high-density task arrival scenarios.

## 6. Conclusions

In this paper, we have studied the joint resource allocation and DNN model partitioning issue in UAV-assisted MEC systems and have designed a joint optimization framework by leveraging the A3C-PPO approach. First, we have formulated the DNN partitioning as a decision process. The framework utilizes the PPO algorithm to learn the optimal partitioning points of complex DNN structures to alleviate the computational burden on resource-constrained UAVs. Second, the framework utilizes the asynchronous multi-threaded architecture of A3C to adapt to dynamic fluctuations in terminal access, thereby optimizing bandwidth allocation efficiency. Furthermore, to minimize task execution latency and ensure fairness among urgent tasks, the framework introduces a weighted priority scheduling algorithm based on computation time and remaining time. The proposed method has consistently outperformed its counterparts in simulations, excelling in average task completion rates, reduced processing latency, and higher resource utilization. It also demonstrates good adaptability to the time-varying task requirements in SAGSIN scenarios. In the future, we will consider extending the studied approach to scenarios involving waveforms in the orthogonal time-frequency space domain to further analyze the Doppler effect issues caused by UAVs. 

## Figures and Tables

**Table 1:** Parameter Symbols.

Parameters	Meaning
M={0,1,…,M}	Number of UAVs
N={0,1,…,N}	Number of intelligent terminals
$U=\{u_{1},u_{2},\ldots ,u_{M}\}$	Set of UAVs
$V=\{v_{1},v_{2},\ldots ,v_{N}\}$	Set of intelligent terminals
$j_{i}$	DNN inference task running on intelligent terminal $v_{i}$
$L_{i}$	Total number of layers in the DNN model for task $j_{i}$
$T_{i,tot}^{k}$	Total execution latency of task $j_{i}$ at partitioning point *k*
$T_{i,ed}^{k,q}$	Execution latency of the first *k* layers on the ED (including queuing latency)
$T_{i,ed}^{k}$	Pure computation latency of the first *k* layers on the ED
$T_{i,ed}^{q}$	Queuing waiting latency on the ED
$T_{i,tran}^{k}$	Transmission latency of the *k*-th layer data from ED to UAV
$T_{i,uav}^{k}$	Execution latency of the remaining $L_{i}-k$ layers on the UAV
$d_{i,m}$	Distance between UAV $u_{m}$ and intelligent terminal $v_{n}$
$g_{i,m}$	Channel power gain between UAV $u_{m}$ and intelligent terminal $v_{n}$
$b_{i,m}$	Bandwidth allocated by UAV $u_{m}$ to intelligent terminal $v_{n}$
$B_{m}$	Total available bandwidth of UAV $u_{m}$
$P_{i,m}$	Transmission power from intelligent terminal $v_{n}$ to UAV $u_{m}$
$N_{0}$	Noise power spectral density at the UAV
$D_{i}^{k}$	Data size offloaded at the *k*-th layer of task $j_{i}$
$u_{m}$	Maximum number of intelligent terminals that UAV $u_{m}$ can serve
$T_{i}^{max}$	Maximum tolerable latency for task $j_{i}$

**Table 2:** Simulation Parameter Settings.

Parameter Description	Setting
A3C discount factor γ	0.9
Asynchronous Advantage Actor-Critic network learning rate	$1\times 10^{-5}$, $5\times 10^{-6}$, $1\times 10^{-6}$
Parameter synchronization steps between global neural network and worker neural network	3
PPO discount factor γ	0.9
PPO GAE decay coefficient λ (Generalized Advantage Estimation)	0.95
PPO clipping parameter ϵ (clip_epsilon)	0.2
Weighting coefficients α, β, γ of weighted priority scheduling algorithm	0.5, 0.25, 0.25
Decay coefficient λ of weighted priority scheduling algorithm	2
Total available bandwidth ω of UAV	20–60 MHz
Noise power spectral density $N_{0}$ between UAVs	−170 dBm/Hz
Channel power gain $\beta_{0}$ at 1-m distance	−20 dB

**Table 3:** ResNet18 Partitioning Positions at Different Cutting Points.

Position	DNN Part Executed by Intelligent Terminal	DNN Part Executed by UAV	Data Size (bit)
1	None	All	1,048,576
2	Conv + Batch Normalization + ReLU	MaxPool + Conv2 to Conv5 + AvgPool + FC Layer	2,097,152
3	Conv + Batch Normalization + ReLU + MaxPool	Conv2 to Conv5 + AvgPool + FC Layer	524,288
4	Conv + Batch Normalization + ReLU + MaxPool + Conv2	Conv3 + Conv4 + Conv5 + AvgPool + FC Layer	524,288
5	Conv + Batch Normalization + ReLU + MaxPool + Conv2 + Conv3	Conv4 + Conv5 + AvgPool + FC Layer	262,144
6	Conv + Batch Normalization + ReLU + MaxPool + Conv2 + Conv3 + Conv4	Conv5 + AvgPool + FC Layer	131,072
7	Conv + Batch Normalization + ReLU + MaxPool + Conv2 to Conv5	AvgPool + FC Layer	65,536
8	Conv + Batch Normalization + ReLU + MaxPool + Conv2 to Conv5 + AvgPool	FC Layer	16,384
9	All	None	0

## Data Availability

Data available on request due to restrictions.
